# Supplementary material for: Multi‐dataset identification of innovative feature genes and molecular mechanisms in keratoconus
Source: J Cell Mol Med. 2024 Sep 19;28(18):e70079. doi: 10.1111/jcmm.70079 (PMC11412914; doi:10.1111/jcmm.70079)
Supplement: Supplementary file 2 — Material S2. [file JCMM-28-e70079-s002.zip › 7.GSEA analysis/5.RNF217-AS1/KEGG_N_GLYCAN_BIOSYNTHESIS.html]

Details for gene set KEGG\_N\_GLYCAN\_BIOSYNTHESIS[GSEA]

|  || Dataset | RNF217-AS1.group.cls#h\_versus\_l |
| Phenotype | group.cls#h\_versus\_l |
| Upregulated in class | l |
| GeneSet | KEGG\_N\_GLYCAN\_BIOSYNTHESIS |
| Enrichment Score (ES) | -0.42107433 |
| Normalized Enrichment Score (NES) | -1.3878757 |
| Nominal p-value | 0.19348268 |
| FDR q-value | 0.3799942 |
| FWER p-Value | 0.884 |
Table: GSEA Results Summary

  

Fig 1: Enrichment plot: KEGG\_N\_GLYCAN\_BIOSYNTHESIS      
 Profile of the Running ES Score & Positions of GeneSet Members on the Rank Ordered List

  

| SYMBOL | TITLE | RANK IN GENE LIST | RANK METRIC SCORE | RUNNING ES | CORE ENRICHMENT || 1 | MAN1B1 | na | 8706 | 0.112 | -0.2218 | No |
| 2 | DPM3 | na | 10264 | 0.082 | -0.2453 | No |
| 3 | B4GALT1 | na | 10311 | 0.081 | -0.2254 | No |
| 4 | MGAT5 | na | 12048 | 0.055 | -0.2611 | No |
| 5 | STT3B | na | 12291 | 0.051 | -0.2546 | No |
| 6 | MGAT4B | na | 12958 | 0.043 | -0.2626 | No |
| 7 | ALG13 | na | 14204 | 0.028 | -0.2911 | No |
| 8 | RPN1 | na | 14849 | 0.021 | -0.3043 | No |
| 9 | B4GALT2 | na | 15023 | 0.019 | -0.3044 | No |
| 10 | MOGS | na | 15172 | 0.017 | -0.3041 | No |
| 11 | ALG11 | na | 15710 | 0.011 | -0.3166 | No |
| 12 | GANAB | na | 15979 | 0.009 | -0.3219 | No |
| 13 | MAN1A2 | na | 16077 | 0.008 | -0.3227 | No |
| 14 | STT3A | na | 17052 | -0.002 | -0.3502 | No |
| 15 | DAD1 | na | 17180 | -0.003 | -0.3530 | No |
| 16 | DDOST | na | 17416 | -0.006 | -0.3583 | No |
| 17 | ALG3 | na | 19003 | -0.020 | -0.3988 | No |
| 18 | RPN2 | na | 19452 | -0.025 | -0.4052 | No |
| 19 | DPM2 | na | 19802 | -0.028 | -0.4079 | No |
| 20 | ALG12 | na | 20160 | -0.031 | -0.4100 | Yes |
| 21 | DPM1 | na | 20195 | -0.032 | -0.4026 | Yes |
| 22 | ALG5 | na | 20326 | -0.033 | -0.3977 | Yes |
| 23 | ST6GAL1 | na | 20869 | -0.038 | -0.4032 | Yes |
| 24 | ALG8 | na | 20879 | -0.039 | -0.3934 | Yes |
| 25 | ALG14 | na | 21289 | -0.043 | -0.3939 | Yes |
| 26 | MGAT3 | na | 22232 | -0.053 | -0.4072 | Yes |
| 27 | MGAT1 | na | 22410 | -0.055 | -0.3979 | Yes |
| 28 | B4GALT3 | na | 22689 | -0.058 | -0.3906 | Yes |
| 29 | ALG2 | na | 22765 | -0.059 | -0.3774 | Yes |
| 30 | ALG6 | na | 22861 | -0.060 | -0.3644 | Yes |
| 31 | MGAT4A | na | 24381 | -0.081 | -0.3870 | Yes |
| 32 | DOLPP1 | na | 24629 | -0.085 | -0.3720 | Yes |
| 33 | MAN1A1 | na | 25563 | -0.099 | -0.3729 | Yes |
| 34 | ALG9 | na | 26304 | -0.112 | -0.3649 | Yes |
| 35 | ALG10B | na | 26505 | -0.116 | -0.3402 | Yes |
| 36 | MGAT2 | na | 26752 | -0.121 | -0.3156 | Yes |
| 37 | TUSC3 | na | 26873 | -0.123 | -0.2869 | Yes |
| 38 | DPAGT1 | na | 27954 | -0.147 | -0.2795 | Yes |
| 39 | ALG1 | na | 28218 | -0.154 | -0.2468 | Yes |
| 40 | RFT1 | na | 28354 | -0.157 | -0.2095 | Yes |
| 41 | ALG10 | na | 29595 | -0.194 | -0.1945 | Yes |
| 42 | MAN1C1 | na | 30106 | -0.210 | -0.1543 | Yes |
| 43 | MAN2A2 | na | 30985 | -0.241 | -0.1165 | Yes |
| 44 | MGAT5B | na | 31437 | -0.260 | -0.0615 | Yes |
| 45 | MAN2A1 | na | 31576 | -0.267 | 0.0043 | Yes |
| 46 | FUT8 | na | 32592 | -0.331 | 0.0616 | Yes |
Table: GSEA details [plain text format]

  

Fig 2: KEGG\_N\_GLYCAN\_BIOSYNTHESIS      
 Blue-Pink O' Gram in the Space of the Analyzed GeneSet

  

Fig 3: KEGG\_N\_GLYCAN\_BIOSYNTHESIS: Random ES distribution      
 Gene set null distribution of ES for **KEGG\_N\_GLYCAN\_BIOSYNTHESIS**
